# Toll-Like Receptor 3 Signal in Dendritic Cells Benefits Cancer Immunotherapy

**DOI:** 10.3389/fimmu.2017.01897

**Published:** 2017-12-21

**Authors:** Misako Matsumoto, Yohei Takeda, Megumi Tatematsu, Tsukasa Seya

**Affiliations:** ^1^Department of Vaccine Immunology, Hokkaido University Graduate School of Medicine, Sapporo, Japan

**Keywords:** adjuvant, cancer immunotherapy, checkpoint inhibitors, cross-priming, dendritic cells, double-stranded RNA, innate immunity, toll-like receptor 3

## Abstract

Pattern recognition receptors (PRRs) play a crucial role in the innate immune system and contribute to host defense against microbial infection. PRR-mediated antimicrobial signals provide robust type-I IFN/cytokine production and trigger inflammation, thereby affecting tumor progression and autoimmune diseases. Accumulating evidence demonstrates that among the PRRs, only the signaling pathway of endosomal toll-like receptor 3 (TLR3) induces no systemic inflammation and mediates cross-priming of antigen-specific CD8^+^ T cells by dendritic cells. Treatment with a newly developed TLR3-specific ligand, ARNAX, along with tumor-associated antigens (TAAs), induces tumor-specific cytotoxic T lymphocytes, modulates the tumor microenvironment to establish Th1-type antitumor immunity, and leads to tumor regression without inflammation in mouse tumor models. Combination therapy using ARNAX/TAA and PD-1/PD-L1 blockade potently enhances antitumor response and overcomes anti-PD-1/PD-L1 resistance. In this review, we will discuss the TLR3-mediated signaling in antitumor immunity and its application to cancer immunotherapy.

## Introduction

The innate immune system senses pathogen- and host-derived nucleic acids to maintain host homeostasis (1). Nucleic-acid-sensing innate immune receptors can be classified into two groups: (1) direct antiviral receptors that induce robust type-I IFN/cytokine production (2–4) and (2) dendritic cell (DC)-priming receptors that induce adaptive immunity (5, 6). The former consists of endosomal toll-like receptor (TLR) 7, 8, and 9, cytoplasmic RNA sensors retinoic acid-inducible gene-I (RIG-I), and melanoma differentiation-associated protein 5 (MDA5) and DNA sensors cGAS and STING, whose activation is closely associated with systemic IFN/cytokinemia and autoimmune diseases resulting from the recognition of endogenous RNA/DNA (7–9). The resultant IFN/cytokines induce inflammation and trans-activate antigen (Ag)-presenting DCs. On the other hand, TLR3 belongs to the latter group due to its restricted expression in myeloid DCs and usage of the signaling adaptor protein toll-IL-1 receptor-containing adaptor molecule-1 (TICAM-1) (also named TRIF) (10). The TLR3–TICAM-1 pathway predominantly works in professional Ag-presenting DCs to cross-prime CD8^+^ T cells as well as to induce production of Th1-type cytokines/chemokines (11, 12). However, polyinosinic:polycytidylic acid [poly(I:C)] that activates both TLR3 and MDA5 has been used as a TLR3 ligand. Studies with a recently developed TLR3-specific agonist, ARNAX, demonstrate that TLR3–TICAM-1 signaling primarily induces DC-priming without systemic cytokine production (13–15). The results suggest that TLR3-specific signal is non-inflammatory and RNA-driven inflammation is rooted in the systemic cytoplasmic pathway (Table 1). Hence, in the context of DC-priming, targeting endosomal TLR3 is a promising strategy for induction of antitumor immunity.

**Table 1:** Nucleic-acid-sensing innate immune receptors.

Receptor	Ligand	Signaling adaptor	Localization	Cell	Function	Reference
TLR3	Viral dsRNA, virus/host structured ssRNA, Poly(I:C), ARNAX	TICAM-1 (localization: cytoplasm)	Endosome	Myeloid DC, macrophage, fibroblast, epithelial cell	Antiviral, NK activation, CTL induction, IgA production	(5, 6, 10, 11, 13)
TLR7	Virus/host ssRNA, imidazoquinoline compound	MyD88 (localization: cytoplasm)	Endosome	Plasmacytoid DC, B cell	Antiviral (type-I IFN), Ab production	(2, 7)
TLR8	Virus/host ssRNA, imidazoquinoline compound	MyD88 (localization: cytoplasm)	Endosome	Myeloid DC, monocyte, neutrophil	Antiviral, inflammatory cytokine production	(2, 7)
TLR9	CpG DNA, chromatin/DNA complex	MyD88 (localization: cytoplasm)	Endosome	Plasmacytoid DC, B cell	Antiviral (type-I IFN), Ab production	(2, 7)
RIG-I	Viral 5′ppp-dsRNA	MAVS (localization: mitochondrion)	Cytoplasm	Ubiquitous	Antiviral (type-I IFN) inflammatory cytokine production	(2, 3, 8)
MDA5	Viral long dsRNA, Poly(I:C)	MAVS (localization: mitochondrion)	Cytoplasm	Ubiquitous	Antiviral (type-I IFN), inflammatory cytokine production, NK activation	(2, 3, 8)
cGAS	dsDNA	STING (localization: endoplasmic reticulum)	Cytoplasm	Ubiquitous	Antiviral (type-I IFN), inflammatory cytokine production, CTL induction	(4, 9)
STING	Cyclic dinucleotide		Endoplasmic reticulum	Ubiquitous	Antiviral (type-I IFN), inflammatory cytokine production, CTL induction	(4, 9)

Toll-like receptor 3 is expressed on endosomal membranes in myeloid DCs, as well as on both cell and endosomal membranes in macrophages, fibroblasts, and some kinds of epithelial cells (16). Professional Ag-presenting DCs, including mouse CD8α^+^ and CD103^+^ DCs as well as human CD141^+^ DCs, highly express TLR3 (17, 18). TLR3 recognizes virus-derived double-stranded RNA (dsRNA) and virus- or host-derived single-stranded RNA having incomplete stem structures (19). Upon ligand recognition within endosomes, TLR3 oligomerizes and recruits the adaptor molecule TICAM-1, which activates the transcription factors NF-κB, IRF3, and AP-1, leading to the production of cytokines (IL-6, TNF-α, IL-12) and type-I IFN (especially IFN-β) (20, 21). TBK-1 is critical for IRF3 activation in the TICAM-1 signalosome, which resides in perinuclear regions as speckles following the dissociation from endosomal TLR3 (22). TICAM-1 is expressed in various cells and tissues at low levels, and TICAM-1 activation is tightly regulated by the conformational context of protein–protein associations (23). Spatiotemporal regulation of the TLR3–TICAM-1 pathway might be important for triggering non-inflammation and Th1-type adaptive immune responses.

## ARNAX Signaling Pathway

**Figure 1:**
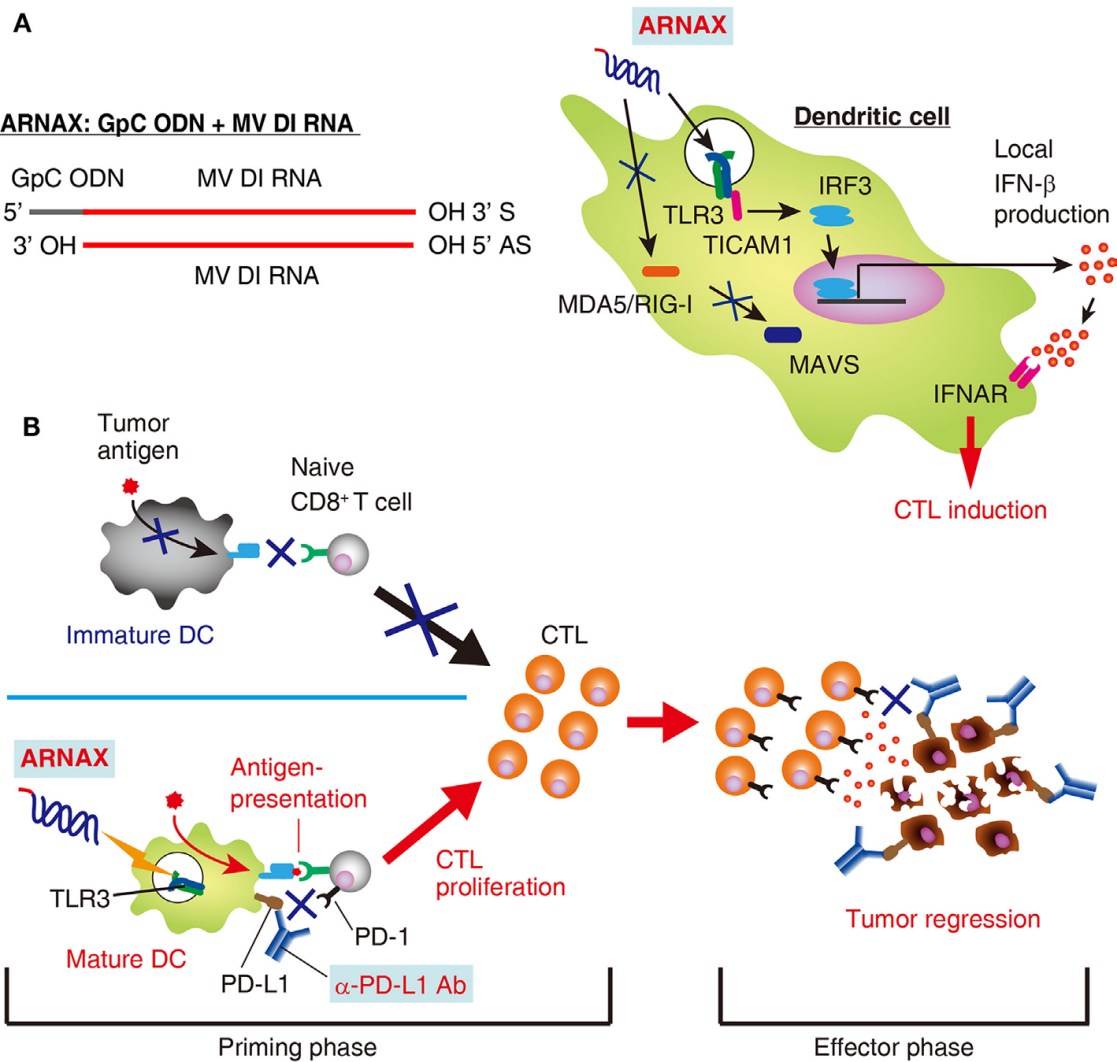
Structure and function of ARNAX. **(A)** Structure and signaling pathway of ARNAX. ARNAX activates endosomal toll-like receptor 3 (TLR3), but not cytoplasmic MDA5/RIG-I. The TLR3–TICAM-1–IRF3–IFN-β signaling axis is indispensable in dendritic cells (DCs) for ARNAX-mediated cytotoxic T lymphocyte (CTL) induction. **(B)** ARNAX therapy enhances antitumor responses in conjunction with PD-1/PD-L1 blockade. Tumors are self-originating and essentially lack adjuvant. In the absence of adjuvant, DCs remain immature state (immature DC) and fail to induce tumor-associated antigen (TAA)-specific CTLs (upper left panel). ARNAX activates TLR3 in DCs to induce maturation and cross-priming of TAA-specific CTLs in lymphoid tissues (priming phase) (lower left panel). PD-1/PD-L1 blockade potentiates ARNAX-mediated CTL induction in the priming phase and reinvigorates tumor infiltrating CTLs in the effector phase (right panel).

ARNAX is a synthetic DNA–dsRNA hybrid molecule consisting of 140 bp of measles virus vaccine strain-derived dsRNA with a 5′ GpC-type phosphorothioated oligodeoxynucleotides (ODNs) cap (Figure 1A) (13, 24). DNA–RNA conjunction sites and dsRNA regions are relatively resistant to nucleases (25) and measles virus-derived dsRNA fails to induce RNA interference against host cell-derived RNAs, suggesting a stable and safer structure. The GpC ODN cap guides dsRNA to TLR3-positive cells for endocytosis, where the dsRNA activates TLR3 (26). dsRNA with a length of >90 bp is required for sufficient activation and signal transduction of the TLR3–TICAM-1 pathway (18). These ARNAX structural motifs do not stimulate cytoplasmic RNA sensors, RIG-I and MDA5, or DNA sensors: they are ubiquitously expressed all over the body.

The viral dsRNA analogue poly(I:C)—a well-known ligand for TLR3—additionally activates the cytoplasmic dsRNA sensor MDA5 (27, 28), resulting in systemic and robust production of type-I IFNs/cytokines that causes undesirable inflammation. In contrast to poly(I:C), ARNAX induces marginal inflammatory cytokine/IFN-β production in a TLR3–TICAM-1-dependent fashion, demonstrating that the TLR3–TICAM-1 pathway contributes to minimal and local cytokine release to effectively prime DCs. Furthermore, the Th1-type cytokine IL-12 is substantially induced by ARNAX, as per poly(I:C), in a TLR3-dependent manner upon subcutaneous injection in mice (29). Non-inflammatory features of ARNAX and its Th1-skewing profile can be attributed to the restricted expression profile and early endosome localization of TLR3 as well as the TICAM-1 signalosome composition.

ARNAX activates professional Ag-presenting DCs to cross-prime CD8^+^ T cells. The TLR3–TICAM-1–IRF3–IFN-β signaling axis in DCs is indispensable for ARNAX-induced Ag-specific CD8^+^ T-cell priming (Figure 1A) (15). MAVS (signaling adaptor of RIG-I-like receptors), MyD88 (adaptor of all TLRs except for TLR3), and STING (adaptor of DNA sensors) do not contribute to ARNAX-induced cross-priming of CD8^+^ T cells (30). Importantly, DC-mediated local, but not systemic, IFN-β production is sufficient for CD8^+^ T-cell cross-priming (15, 31, 32), although the molecular determinants that regulate cross-priming downstream of the IFN-α/β receptor remains undefined. Thus, ARNAX is a non-inflammatory DC-priming adjuvant that specifically targets the TLR3–TICAM-1 pathway.

## Induction of Antitumor Immunity

The presence of functional tumor-specific cytotoxic T lymphocytes (CTLs) in the tumor microenvironment is mandatory for tumor regression (33). ARNAX can induce tumor-specific CTLs by activating Ag-presenting DCs. In mouse implant tumor models EG7 and MO5 (OVA expressing T lymphoma and melanoma, respectively), injection of ARNAX along with OVA regresses tumor growth (15). OVA-specific CD8^+^ T cells proliferate in both lymphoid tissues and within tumors. Furthermore, CD8α^+^ DCs and CD8^+^ T cells increasingly infiltrate into tumor. Numerous genes associated with antitumor immunity are significantly upregulated in whole EG7 tumors during the ARNAX + OVA therapy (15). Chemokine genes responsible for recruiting DCs and T cells (*Ccl4, Ccl5, Ccl27*) (34), NK/T-cell function-related genes (*Gzmb, Prf1, Fasl*), cell adhesion-related genes, and cytokine receptor genes such as *IL2rb* and *IL12rb1* are also upregulated. ARNAX and TAA therapy thus fosters Th1-type antitumor immunity in these tumor models. Hence, vaccine immunotherapy with TLR3 adjuvant enables to establish antitumor immunity against certain tumor types.

Notably, ARNAX monotherapy induces tumor growth retardation (15). It is likely that DCs internalize tumor cell debris, which contains TAAs, and cross-prime CD8^+^ T cells *via* TLR3-mediated activation of DCs (35). Alternatively, TLR3 signaling may facilitate the infiltration of preexisting tumor-reactive CTLs into tumor sites by inducing chemokine production. Mouse CD8α^+^ DCs and human CD141^+^ DCs express the C-type lectin-like receptor CLEC9A on their cell surface, which is involved in phagocytosis of dead cell debris that contains TAAs (36, 37). The quality of TAAs and their efficient delivery to DCs are important factors influencing the validation of vaccine immunotherapy with TLR3 adjuvant.

## Modulation of the Tumor Microenvironment

The tumor microenvironment strongly affects tumor progression and antitumor immunity (38–40). Tumor-associated macrophages (TAMs), granulocytic or monocytic myeloid-derived suppressor cells (G- or M-MDSCs), and regulatory T cells are major constituents of the immunosuppressive tumor microenvironment (41, 42). The extent and composition of immune cell infiltration within tumors considerably differ among tumor types (42, 43). Accumulating evidence suggests that modulation of the tumor microenvironment from immunosuppressive to immunosupportive is a crucial factor for the success of cancer immunotherapy (44–46). It has emerged from several studies that TLR activation changes the properties of the tumor microenvironment (47–49). Among the TLR ligands, TLR2 ligand enhances the survival of M-MDSCs and their differentiation into macrophages, which augments the immunosuppressive activity of M-MDSCs toward CD8^+^ T cells through iNOS expression from macrophages (49). In contrast, TLR3 ligand converts TAMs from an M2- to an M1-like phenotype (47). When activated with poly(I:C), TAMs robustly produced TNF-α in 3LL (mouse lung carcinoma cell line) tumor in mice, resulting in tumor cell death and growth suppression. The TLR3–TICAM-1 pathway is critical for poly(I:C)-induced tumor regression via stromal macrophages in the 3LL tumor mouse model. In addition, G-MDSCs act as effector but not suppressor cells upon activation with TLR3 ligand in the EL4 tumor model (48). G-MDSCs produce reactive oxygen species through the TLR3–TICAM-1 pathway, leading to tumor growth inhibition (48). Furthermore, the proportion of G-MDSCs in EG7 tumors is greatly decreased by ARNAX + TAA therapy (15), which makes CTL unexhausted. Thus, TLR3 signaling functionally ameliorates the tumor microenvironment to potentiate antitumor immunity.

## Combination Therapy of ARNAX with Anti-PD-1/PD-L1 Antibodies

Recent advances in cancer immunotherapy with checkpoint inhibitors have shown durable antitumor responses and good prognoses in patients with melanoma and non-small cell lung cancer, but only ~20% of patients with solid tumors respond to checkpoint blockade (50–54). The presence of preexisting tumor-specific CD8^+^ T cells and their infiltration into tumor sites are required for responsiveness to PD-1/PD-L1 blockade therapy (51). Many cancer patients, however, have limited numbers of tumor-specific CTLs, if at all, as well as/or the presence of therapy-resistant tumor microenvironments (55). The appearance and amount of mutation-associated neo-antigens in tumors correlates with sensitivity to PD-1 blockade (56–58), which is likely associated with preexisting tumor-specific CTLs (59). However, molecular determinants of tumor cells that define CTL induction by the immune system are still unclear. In this setting, vaccine immunotherapy that potently induces tumor-specific CD8^+^ T cells through DC-priming is a feasible approach to overcome primary resistance to PD-1/PD-L1 blockade.

In anti-PD-L1 antibody unresponsive mouse tumors, vaccine immunotherapy using ARNAX and tumor Ag decreases tumor progression irrespective of PD-L1 levels on tumor cells (15). Combination therapy with anti-PD-L1 antibody and ARNAX + TAA induces an antitumor response more effectively than anti-PD-L1 antibody monotherapy, especially in tumors expressing high levels of PD-L1. Priming of tumor-specific CD8^+^ T cells in lymphoid tissues and the infiltration of CD8^+^ T cells into the tumor site are greatly enhanced by combination therapies. DCs and macrophages in lymphoid tissues express PD-L1 at low or intermediate levels (60, 61). It is conceivable that blockade of the PD-1/PD-L1 pathway augments TAA-specific CD8^+^ T-cell induction from ARNAX + TAA therapy at the priming phase, and infiltrated CTLs are reinvigorated at the effector phase (62, 63) (Figure 1B). Tumor-associated DCs and CD11b^+^ immunosuppressive myeloid cells express high levels of PD-L1 within tumors (60), which is independent of efficacy of ARNAX + TAA therapy in several mouse tumor models. Although PD-L1 levels on tumor cells and infiltrated myeloid cells are one of the predictive biomarkers for responsiveness to PD-1/PD-L1 blockade (64, 65), tumor PD-L1 level is not always applicable for a prognostic biomarker to the ARNAX therapy. Potent induction of tumor-specific CD8^+^ T cells with DC-priming adjuvant and PD-1/PD-L1 blockade is important for infiltration and reinvigoration of CD8^+^ T cells within tumors.

## Conclusion

To overcome the unresponsiveness of tumors to anti-PD-1/PD-L1 therapy, many cancer immunotherapy approaches have been conducted. Vaccine immunotherapy with TAA and the DC-priming adjuvant ARNAX generates tumor-specific CTLs with minimal essential cytokine production, which appears to avoid exacerbating adverse effects observed in certain population of patients treated with checkpoint inhibitors, such as the onset of autoimmune diseases. Up until now, numerous trials of peptide vaccine therapies have been performed unsuccessfully (66). The main factor contributing to the ineffectiveness is thought to be the usage of inflammatory, as opposed to DC-priming, adjuvants in peptide vaccine therapies. On the other hand, several preclinical studies with vaccine immunotherapies using poly(I:C) as a DC-priming adjuvant have been approved (67–70). However, poly(I:C) triggers undesirable inflammation caused by cytokine toxicity (67). The introduction of the non-inflammatory adjuvant ARNAX to peptide vaccine immunotherapy and/or combination therapy with PD-1 blockade appears to be a promising strategy to overcome anti-PD-1 resistance. Notably, induction of tumor cell death by radiation, chemotherapy, and oncolytic viruses appear to liberate TAAs from tumors (71–74), which may cooperate with ARNAX to induce polyclonal tumor-reactive CTLs and facilitate suppression of tumor growth.

In cancer immunotherapy, acquired resistance to newly developed therapies is a subject of intense discussion. In the case of TLR3 adjuvant therapy, the acquisition of resistance to CTL activity by tumor cells should be investigated. Evaluation of the quality and quantity of TAAs in individual tumors could be a therapeutic prerequisite for TLR3 adjuvant therapy, but this prediction has not been confirmed. Further studies elucidating the mechanism of tumor evasion from CTL cytotoxicity and the development of appropriate protocols for TLR3 adjuvant therapy would prove useful in this field to allow for complete tumor regression in cancer patients.

## Author Contributions

MM, YT, MT, and TS conceived and designed the experiments. YT and MT performed the experiments. MM and TS wrote the paper.

## Conflict of Interest Statement

The authors declare that the research was conducted in the absence of any commercial or financial relationships that could be construed as a potential conflict of interest.

## References

[1] Desmet CJ Ishii KJ. Nucleic acid sensing at the interface between innate and adaptive immunity in vaccination. Nat Rev Immunol (2012) 12:479–91. 10.1038/nri3247 22728526

[2] Kawai T Akira S. Innate immune recognition of viral infection. Nat Immunol (2006) 7:131–7. 10.1038/ni1303 16424890

[3] Yoneyama M Onomoto K Jogi M Akaboshi T Fujita T. Viral RNA detection by RIG-I-like receptors. Curr Opin Immunol (2015) 32:48–53. 10.1016/j.coi.2014.12.012 25594890

[4] Chen Q Sun L Chen ZJ. Regulation and function of the cGAS-STING pathway of cytosolic DNA sensing. Nat Immunol (2016) 17:1142–9. 10.1038/ni.3558 27648547

[5] Schulz O Diebold SS Chen M Näslund TI Nolte MA Alexopoulou L Toll-like receptor 3 promotes cross-priming to virus-infected cells. Nature (2005) 433:887–92. 10.1038/nature03326 15711573

[6] Takaki H Kure S Oshiumi H Sakoda Y Suzuki T Ainai A Toll-like receptor 3 in nasal CD103+ dendritic cells is involved in immunoglobulin A production. Mucosal Immunol (2017) 61:107–13. 10.1038/mi.2017.48 28612840

[7] Krieg AM Vollmer J. Toll-like receptors 7, 8, and 9: linking innate immunity to autoimmunity. Immunol Rev (2007) 220:251–69. 10.1111/j.1600-065X.2007.00572.x 17979852

[8] Kato H Fujita T RIG-I-like receptors and autoimmune diseases. Curr Opin Immunol (2015) 37:40–5. 10.1016/j.coi.2015 26530735

[9] Liu Y Jesus AA Marrero B Yang D Ramsey SE Sanchez GAM Activated STING in a vascular and pulmonary syndrome. N Engl J Med (2014) 371:507–18. 10.1056/NEJMoa1312625 25029335 PMC4174543

[10] Matsumoto M Seya T TLR3: interferon induction by double-stranded RNA including poly(I:C). Adv Drug Del Rev (2008) 60:805–12. 10.1016/j.addr.2007.11.005 18262679

[11] Azuma M Ebihara T Oshiumi H Matsumoto M Seya T. Cross-priming for antitumor CTL induced by soluble Ag + polyI:C depends on the TICAM-1 pathway in mouse CD11c(+)/CD8α(+) dendritic cells. Oncoimmunology (2012) 1:581–92. 10.4161/onci.19893 22934250 PMC3429562

[12] Azuma M Takeda Y Nakajima H Sugiyama T Ebihara T Oshiumi H Biphasic function of TLR3 adjuvant on tumor and spleen dendritic cells promotes tumor T cell infiltration and regression in a vaccine model. Oncoimmunology (2016) 5:e1188244 10.1080/2162402X.2016.1188244 27622060 PMC5007976

[13] Matsumoto M Tatematsu M Nishikawa F Azuma M Ishii N Morii-Sakai A Defined TLR3-specific adjuvant that induces NK and CTL activation without significant cytokine production in vivo. Nat Commun (2015) 6:6280. 10.1038/ncomms7280 25692975

[14] Seya T Takeda Y Matsumoto M. Tumor vaccines with dsRNA adjuvant ARNAX induces antigen-specific tumor shrinkage without cytokinemia. Oncoimmunology (2015) 5:e1043506. 10.1080/2162402X.2015.1043506 27057425 PMC4801468

[15] Takeda Y Kataoka K Yamagishi J Ogawa S Seya T Matsumoto M A TLR3-specific adjuvant relieves innate resistance to PD-L1 blockade without cytokine toxicity in tumor vaccine immunotherapy. Cell Rep (2017) 19:1874–87. 10.1016/j.celrep.2017.05.015 28564605

[16] Matsumoto M Funami K Tanabe M Oshiumi H Shingai M Seto Y Subcellular localization of toll-like receptor 3 in human dendritic cells. J Immunol (2003) 171:3154–62. 10.4049/jimmunol.171.9.4934-b 12960343

[17] Jongbloed SL Kassianos AJ McDonald KJ Clark GJ Ju X Angel CE Human CD141+ (BDCA-3)+ dendritic cells (DCs) represent a unique myeloid DC subset that cross-presents necrotic cell antigens. J Exp Med (2010) 207:1247–60. 10.1084/jem.20092140 20479116 PMC2882828

[18] Jelinek I Leonard JN Price GE Brown KN Meyer-Manlapat A Goldsmith PK TLR3-specific double-stranded RNA oligonucleotide adjuvants induce dendritic cell cross-presentation, CTL responses, and antiviral protection. J Immunol (2011) 186:2422–9. 10.4049/jimmunol.1002845 21242525 PMC3078629

[19] Tatematsu M Nishikawa F Seya T Matsumoto M. Toll-like receptor 3 recognizes incomplete stem structures in single-stranded viral RNA. Nat Commun (2013) 4:1833. 10.1038/ncomms2857 23673618

[20] Oshiumi H Matsumoto M Funami K Akazawa T Seya T TICAM-1, an adaptor molecule that participates in toll-like receptor 3-mediated interferon β-induction. Nat Immunol (2003) 4:161–7. 10.1038/ni886 12539043

[21] Yamamoto M Sato S Hemmi H Uematsu S Hoshino K Kaisho T Role of adaptor TRIF in the MyD88-independent toll-like receptor signaling pathway. Science (2003) 301:640–3. 10.1126/science.1087262 12855817

[22] Funami K Sasai M Ohba Y Oshiumi H Seya T Matsumoto M Spatiotemporal mobilization of TICAM-1 in response to dsRNA. J Immunol (2007) 179:6867–72. 10.4049/jimmunol.179.10.6867 17982077

[23] Tatematsu M Ishii A Oshiumi H Horiuchi M Inagaki F Seya T A molecular mechanism for Toll/IL-1 receptor domain-containing adaptor molecule-1-mediated IRF-3 activation. J Biol Chem (2010) 285:20128–36. 10.1074/jbc.M109.099101 20418377 PMC2888425

[24] Shingai M Ebihara T Begum NA Okabe M Akazawa T Miyamoto Y Differential type I interferon (IFN) inducing abilities of wild-type vs. vaccine strains of measles virus. J Immunol (2007) 179:6123–33. 10.4049/jimmunol.179.9.6123 17947687

[25] Mochizuki S Higuchi S Sakurai K ssDNA–dsRNAs are cleaved at the next to its chimera-junction point by an unknown RNase activity. Biochem Biophys Res Commun (2012) 428:433–7. 10.1016/j.bbrc.2012.10.100 23131557

[26] Itoh K Watanabe A Funami K Seya T Matsumoto M. The clathrin-mediated endocytic pathway participates in dsRNA-induced IFN-beta production. J Immunol (2008) 181:5522–9. 10.4049/jimmunol.181.8.5522 18832709

[27] Kato H Takeuchi O Sato S Yoneyama M Yamamoto M Matsui K Differential roles of MDA5 and RIG-I helicases in the recognition of RNA viruses. Nature (2006) 441:101–5. 10.1038/nature04734 16625202

[28] Gitlin L Barchet W Gilfillan S Cella M Beutler B Flavell RA Essential role of mda-5 in type I IFN responses to polyriboinosinic:polyribocytidylic acid and encephalomyocarditis picornavirus. Proc Natl Acad Sci U S A (2006) 103:8459–64. 10.1073/pnas.0603082103 16714379 PMC1464000

[29] Gerner MY Heltemes-Harris LM Fife BT Mescher MF. Cutting edge: IL-12 and type I IFN differentially program CD8 T cells for programmed death 1 re-expression levels and tumor control. J Immunol (2013) 191:1011–5. 10.4049/jimmunol.1300652 23804712 PMC3720703

[30] Zevini A Olagnier D Hiscott J. Crosstalk between cytoplasmic RIG-I and STING sensing pathways. Trends Immunol (2017) 38:194–205. 10.1016/j.it.2016.12.004 28073693 PMC5329138

[31] Fuertes MB Kacha AK Kline J Woo SR Kranz DM Murphy KM Host type I IFN signals are required for antitumor CD8+ T cell responses through CD8α+ dendritic cells. J Exp Med (2011) 208:2005–16. 10.1084/jem.20101159 21930765 PMC3182064

[32] Pantel A Teixeira A Haddad E Wood EG Steinman RM Longhi MP. Direct type I IFN but not MDA5/TLR3 activation of dendritic cells is required for maturation and metabolic shift to glycolysis after poly IC stimulation. PLoS Biol (2014) 12:e1001759. 10.1371/journal.pbio.1001759 24409099 PMC3883643

[33] Seya T Matsumoto M. The extrinsic RNA-sensing pathway for adjuvant immunotherapy of cancer. Cancer Immunol Immunother (2009) 58:1175–84. 10.1007/s00262-008-0652-9 19184005 PMC11030714

[34] Bachmann MF Kopf M Marsland BJ Chemokines: more than just road signs. Nat Rev Immunol (2006) 6:159–64. 10.1038/nri1776 16491140

[35] Kroemer G Galluzzi L Keppo O Zitvogel L. Immunogenic cell death in cancer therapy. Annu Rev Immunol (2013) 31:51–72. 10.1146/annurev-immunol-032712-100008 23157435

[36] Poulin LF Salio M Griessinger E Anjos-Afonso F Craciun L Chen JL Characterization of human DNGR-1^+^ BDCA3^+^ leukocytes as putative equivalents of mouse CD8α^+^ dendritic cells. J Exp Med (2010) 207:1261–71. 10.1084/jem.20092618 20479117 PMC2882845

[37] Zhang JG Czabotar PE Policheni AN Caminschi I Wan SS Kitsoulis S The dendritic cell receptor Clec9A binds damaged cells via exposed actin filaments. Immunity (2012) 36:646–57. 10.1042/BJ20121819 22483802

[38] Mantovani A Allavena P Sica A Balkwill F Cancer-related inflammation. Nature (2008) 454:436–44. 10.1038/nature07205 18650914

[39] Coussens LM Zitvogel L Palucks AK Neutralizing tumor-promoting chronic inflammation: a major bullet? Science (2013) 339:286–91. 10.1126/science.1232227 23329041 PMC3591506

[40] Joyce JA Fearon DT T cell exhaustion, immune privilege, and tumor microenvironment. Science (2015) 348:74–9. 10.1126/science.aaa6204 25838376

[41] Peranzoni E Zilio S Marigo I Dolcetti L Zanovello P Mandruzzato S Myeloid-derived suppressor cell heterogeneity and subset definition. Curr Opin Immunol (2010) 22:238–44. 10.1016/j.coi.2010.01.021 20171075

[42] Gajewski TF Schreiber H Fu YX. Innate and adaptive immune cells in the tumor microenvironment. Nat Immunol (2013) 14:1014–22. 10.1038/ni.2703 24048123 PMC4118725

[43] Youn JI Nagaraj S Collazo M Gabrilovich DI. Subsets of myeloid-derived suppressor cells in tumor-bearing mice. J Immunol (2008) 181:5791–802. 10.4049/jimmunol.181.8.5791 18832739 PMC2575748

[44] Gajewski TF Woo SR Zha Y Spaapen R Zheng Y Corrales L Cancer immunotherapy strategies based on overcoming barriers within the tumor microenvironment. Curr Opin Immunol (2013) 25:268–76. 10.1016/j.coi.2013.02.009 23579075

[45] Zelenay S van der Veen AG Bottcher JP Snelgrove KJ Rogers N Acton SE Cyclooxygenase-dependent tumor growth through evasion of immunity. Cell (2015) 162:1257–70. 10.1016/j.cell.2015.08.015 26343581 PMC4597191

[46] De Henau O Rausch M Winkler D Campesato LF Liu C Cymerman DH Overcoming resistance to checkpoint blockade therapy by targeting PI3Kγ in myeloid cells. Nature (2016) 539:443–7. 10.1038/nature20554 27828943 PMC5634331

[47] Shime H Matsumoto M Oshiumi H Tanaka S Nakane A Iwakura Y Toll-like receptor 3 signaling converts tumor-supporting myeloid cells to tumoricidal effectors. Proc Natl Acad Sci U S A (2012) 109:2066–71. 10.1073/pnas.1113099109 22308357 PMC3277567

[48] Shime H Matsumoto M Seya T Double-stranded RNA promotes CTL-independent tumor cytolysis mediated by CD11b^+^Ly6G^+^ intratumor myeloid cells through the TICAM-1 signaling pathway. Cell Death Differ (2016) 24:385–96. 10.1038/cdd.2016.131 27834952 PMC5344202

[49] Shime H Maruyama A Yoshida S Takeda Y Matsumoto M Seya T Toll-like receptor 2 ligand and interferon-γ suppress anti-tumor T cell responses by enhancing the immunosuppressive activity of monocytic myeloid-derived suppressor cells. Oncoimmunology (2017):e1373231 10.1080/2162402X.2017.1373231 PMC5739553 29296526

[50] Hamid O Robert C Daud A Hodi FS Hwu W-J Kefford R Safety and tumor responses with lambrolizumab (anti–PD-1) in melanoma. N Engl J Med (2013) 369:134–44. 10.1056/NEJMoa1305133 23724846 PMC4126516

[51] Tumeh PC Harview CL Yearley JH Shintaku IP Taylor EJ Robert L PD-1 blockade induces responses by inhibiting adaptive immune resistance. Nature (2014) 515:568–71. 10.1038/nature13954 25428505 PMC4246418

[52] Herbst RS Soria JC Kowanetz M Fine GD Hamid O Gordon MS Predictive correlates of response to the anti-PD-L1 antibody MPDL3280A in cancer patients. Nature (2014) 515:563–7. 10.1038/nature14011 25428504 PMC4836193

[53] Sharma P Allison JP. Immune checkpoint targeting in cancer therapy: toward combination strategies with curative potential. Cell (2015) 161:205–14. 10.1016/j.cell.2015.03.030 25860605 PMC5905674

[54] Sharma P Allison JP. The future of immune checkpoint therapy. Science (2015) 348:56–61. 10.1126/science.aaa8172 25838373

[55] Hugo W Zaretsky JM Sun L Song C Moreno BH Hu-Lieskovan S Genomic and transcriptomic features of response to anti-PD-1 therapy in metastatic melanoma. Cell (2016) 165:35–44. 10.1016/j.cell.2016.02.065 26997480 PMC4808437

[56] Rizvi N Hellmann MD Snyder A Kvistborg P Makarov V Havel JJ Mutational landscape determines sensitivity to PD-1 blockade in non-small cell lung cancer. Science (2015) 348:124–8. 10.1126/science.aaa1348 25765070 PMC4993154

[57] Schumacher TN Schreiber RD. Neoantigens in cancer immunotherapy. Science (2015) 348:69–74. 10.1126/science.aaa4971 25838375

[58] Le DT Durham JN Smith KN Wang H Bartlett BR Aulakh LK Mismatch repair deficiency predicts response of solid tumors to PD-1 blockade. Science (2017) 357:409–13. 10.1126/science.aan6733 28596308 PMC5576142

[59] Coulie PG Van den Eynde BJ van der Bruggen P Boon T. Tumour antigens recognized by T lymphocytes: at the core of cancer immunotherapy. Nat Rev Cancer (2014) 5:135–46. 10.1038/nrc3670 24457417

[60] Curiel TJ Wei S Dong H Alvarez X Cheng P Mottram P Blockade of B7-H1 improves myeloid dendritic cell-mediated antitumor immunity. Nat Med (2003) 9:562–7. 10.1038/nm863 12704383

[61] Zou W Wolchok JD Chen L PD-L1 (B7-H1) and PD-1 pathway blockade for cancer therapy: mechanisms, response biomarkers, and combinations. Sci Transl Med (2016) 8:328rv4 10.1126/scitranslmed.aad7118 PMC4859220 26936508

[62] Keir ME Butte MJ Freeman GJ Sharp AH. PD-1 and its ligands in tolerance and immunity. Annu Rev Immunol (2008) 26:677–704. 10.1146/annurev.immunol.26.021607.090331 18173375 PMC10637733

[63] Schietinger A Greenberg PD. Tolerance and exhaustion: defining mechanisms of T cell dysfunction. Trends Immunol (2014) 35:51–60. 10.1016/j.it.2013.10.001 24210163 PMC3946600

[64] Kataoka K Shiraishi Y Takeda Y Sakata S Matsumoto M Nagano S Aberrant PD-L1 expression via 3’-UTR disruption in multiple cancers. Nature (2016) 534:402–6. 10.1038/nature18294 27281199

[65] Kleinovink JW van Hall T Ossendorp F Fransen MF. PD-L1 immune suppression in cancer: tumor cells or host cells? Oncoimmunology (2017) 6:e1325982. 10.1080/2162402X.2017.1325982 28811961 PMC5543902

[66] Rosenberg SA Yang JC Restifo NP. Cancer immunotherapy: moving beyond current vaccines. Nat Med (2004) 10:909–15. 10.1038/nm1100 15340416 PMC1435696

[67] Lampkin BC Levine AS Levy H Krivit W Hammond D Phase II trial of a complex polyriboinosinic-polyribocytidylic acid with poly-L-lysine and carboxymethyl cellulose in the treatment of children with acute leukemia and neuroblastoma: a report from the children’s cancer study group. Cancer Res (1985) 45:5904–9. 2414002

[68] Mbow ML De Gregorio E Valiante NM Rappuoli R. New adjuvants for human vaccines. Curr Opin Immunol (2010) 22:411–6. 10.1016/j.coi.2010.04.004 20466528

[69] Galluzzi L Vacchelli E Eggermont A Fridman WH Galon J Sautès-Fridman C Trial watch: experimental toll-like receptor agonists for cancer therapy. Oncoimmunology (2012) 1:699–716. 10.4161/onci.20696 22934262 PMC3429574

[70] Sabbatini P Tsuji T Ferran L Ritter E Sedrak C Tuballes K Phase I trial of overlapping long peptides from a tumor self-antigen and poly-ICLC shows rapid induction of integrated immune response in ovarian cancer patients. Clin Cancer Res (2012) 18:6497–508. 10.1158/1078-0432.CCR-12-2189 23032745

[71] Deng L Liang H Xu M Yang X Burnette B Arina A STING-dependent cytosolic DNA sensing promotes radiation-induced type I interferon-dependent antitumor immunity in immunogenic tumors. Immunity (2014) 41:843–52. 10.1016/j.immuni.2014.10.019 25517616 PMC5155593

[72] van der Sluis TC van Duikeren S Huppelschoten S Jordanova ES Beyranvand Nejad E Sloots A Vaccine-induced tumor necrosis factor-producing T cells synergize with cisplatin to promote tumor cell death. Clin Cancer Res (2015) 21:781–94. 10.1158/1078-0432.CCR-14-2142 25501579

[73] van der Burg SH Arens R Ossendorp F van Hall T Melief CJ. Vaccines for established cancer: overcoming the challenges posed by immune evasion. Nat Rev Cancer (2016) 16:219–33. 10.1038/nrc.2016.16 26965076

[74] Dovedi SJ Cheadle EJ Popple AL Poon E Morrow M Stewart R Fractionated radiation therapy stimulates antitumor immunity mediated by both resident and infiltrating polyclonal T-cell populations when combined with PD-1 blockade. Clin Cancer Res (2017) 23:5514–26. 10.1158/1078-0432.CCR-16-1673 28533222

